# Persistent polyclonal binucleated B-cell lymphocytosis and MECOM gene amplification

**DOI:** 10.1186/s13104-015-1742-3

**Published:** 2016-03-02

**Authors:** Edouard Cornet, Hossein Mossafa, Karine Courel, Jean-François Lesesve, Xavier Troussard

**Affiliations:** Laboratory of Hematology, Caen University Hospital, Caen, 14000 France; University of Caen, Medical School, Caen, 14000 France; Laboratoire Cerba, Department of Genetic, Saint Ouen L’Aumone, 95310 France; Laboratory of Hematology, Nancy University Hospital, Vandoeuvre-lès-Nancy Cedex, 54511 France

**Keywords:** Persistent Polyclonal Binucleated B-cell Lymphocytosis, MECOM, SNP array

## Abstract

**Background:**

Persistent Polyclonal Binucleated B-cell Lymphocytosis (PPBL) is characterized by a chronic polyclonal B-cell lymphocytosis with binucleated lymphocytes and a polyclonal increase in serum immunoglobulin-M. Cytogenetic is characterized by the presence of a supernumerary isochromosome +i(3)(q10), premature chromosome condensation and chromosomal instability. Outcome of PPBL patients is mostly benign, but subsequent malignancies could occur. The aim of our study is to provide an update of clinical and cytogenetic characteristics of our large cohort of PPBL patients, to describe subsequent malignancies occurring during the follow-up, and to investigate the role of the long arm of chromosome 3 in PPBL.

**Results:**

We analyzed clinical, biological and cytogenetic characteristics (conventional cytogenetic analysis and fluorescent in situ hybridization) of 150 patients diagnosed with PPBL. We performed high-resolution SNP arrays in 10 PPBL patients, comparing CD19^+^ versus CD19^−^ lymphoid cells. We describe the cytogenetic characteristics in 150 PPBL patients consisting in the presence of supernumerary isochromosome +i(3)(q10) (59 %) and chromosomal instability (55 %). In CD19^+^ B-cells, we observed recurrent copy number aberrations of 143 genes with 129 gains (90 %) on 3q and a common minimal amplified genomic region in the *MECOM* gene. After a median follow-up of 60 months, we observed the occurrence of 12 subsequent malignancies (12 %), 6 solid tumors and 6 Non-Hodgkin’s Lymphomas, and 6 monoclonal gammopathies of undetermined significance (MGUS), requiring a long-term clinical follow-up.

**Conclusions:**

Our clinical and cytogenetic observations lead us to hypothesize that isochromosome 3q, especially *MECOM* abnormality, could play a key role in PPBL.

## Background

Persistent Polyclonal Binucleated B-cell Lymphocytosis (PPBL) is characterized by a chronic, stable and asymptomatic lymphocytosis with binucleated lymphocytes [1]. Binucleated lymphocytes are not specific for PPBL and can be observed in patients with multiple sclerosis treated by natalizumab [2] or after accidental exposure to ionizing radiation. In the peripheral blood, a polyclonal increase of memory B cells (CD19^+^, CD5^−^, CD27^+^, IgM^+^, IgD^+^) is usually associated with a polyclonal increase in serum immunoglobulin-M (IgM) [3–6]. PPBL is characterized by a recurrent supernumerary isochromosome +i(3)(q10), a premature chromosome condensation (PCC) and a chromosomal instability [3, 4]. PPBL evolution is benign in most cases, but non-Hodgkin’s lymphomas and solid tumors (pulmonary blastoma) were previously and rarely described [7, 8]. In this study, we report the follow-up and the cytogenetic characteristics of a large cohort of 150 PPBL patients. We report the occurrence of subsequent malignancies in up to 12 % of patients contrasting with previous studies. Strong association between supernumerary isochromosome 3q, chromosomal instability and PPBL led us to study more extensively the role of the long arm of chromosome 3 using SNP arrays in 10 patients. We observed that the *MECOM* gene, located on 3q26, was recurrently amplified in B-cells of PPBL patients.

## Patients and methods

### Patients

PPBL was diagnosed from the persistence during three months of binucleated lymphocytes on a peripheral blood film. Patients were included after written informed consent, in accordance with the Declaration of Helsinki and with institutional guidelines and after approval of the French relevant competent authorities and ethics committees (Committee of Protection of Individuals (CPP), Advisory Committee on the Processing of Information for Medical Research (CCTIRS) and the French National Commission for Data Protection (CNIL)).

Using multiparameter flow cytometry (MFC), B-cells were polyclonal in all cases, based on the expression of CD19 and the absence of a restriction of expression of light chain of immunoglobulin. Blood smears were reviewed in the same laboratory.

### Conventional cytogenetic analysis (CCA)

Blood samples were collected on heparin tubes at the time of diagnosis and during the follow-up. All samples were processed in the same laboratory. CCA was performed as previously described [3]. As previously described [9], chromosomal instability was defined as the gain and/or loss of whole chromosomes or chromosomal segments at a higher rate in tumor cell population compared to normal cells.

### Fluorescent in situ hybridization (FISH)

FISH was performed in order to detect supernumerary isochromosome +i(3)(q10) in metaphase and interphase cells using alpha-satellite chromosome 3 specific probes and Bcl6 (3q27) specific probes (Vysis™, USA). One hundred metaphases and three hundred interphases cells were analyzed per patient.

### SNP array

SNP arrays were performed using Affymetrix™ Cytogenetics Whole-Genome 2.7M Arrays^®^ (Affymetrix™, USA). All samples were processed in the same laboratory. Patients were selected according to the availability of sufficient fresh cells (diagnosis) or frozen cells (follow-up). Immunomagnetic sorting was performed on whole blood samples or on thawed cells in order to purify CD19^+^ cells (Miltenyi™ AutoMACS Pro Separator^®^, Bergisch Gladbach, Germany). The two fractions (CD19^+^ positive and CD19^−^ negative selection) were kept and the purity was checked to be >95 % by flow cytometry. The DNA was extracted from the two fractions using Gentra Puregene Blood Kit^®^ (Qiagen™, Hilden, Germany). Hybridization of the DNA on chips was performed according the manufacturer’s instructions. Chips were analyzed using Affymetrix™ Chromosome Analysis Suite^®^ (ChASver 1.0.1). Database of annotations was NetAffx Build 30. Quality controls of the chips were set up according Affymetrix™ recommendations (SNP-QC ≥ 1.1 and MAPD (CN-QC) ≤ 0.27). Copy Number Aberrations (CNA) were called according user-defined thresholds (Copy Number (CN) markers >50 and size >25 kb). The Database of Genomic Variants (DGV, http://projects.tcag.ca/variation/) was consulted to determine whether CNA corresponded to genomic variants. Number and size of Copy Number Aberrations (CNAs) were analyzed and compared between patients and between CD19^+^ and CD19^−^ cells. CNA are called recurrent when at least two patients present the same CNA. Mosaicism phenomenon was detected in case of allele frequencies between disomic and trisomic states.

## Results

PPBL was diagnosed in 150 untreated patients, whose main characteristics are described in Table 1. Sixty-nine percent of cases showed an absolute lymphocytosis >4 × 10^9^/L, with a mean percentage of binucleated lymphocytes at 3.9 % (1–40). Median follow-up was 60 months (1–402) and median overall survival was not reached. Eighteen patients (12 %) developed subsequent malignancies, among which nine cases were previously described (non Hodgkin’s lymphomas (NHL) in three cases, solid tumors in two cases and monoclonal gammopathies of undetermined significance (MGUS) in 4 cases) [10]. Among the 18 patients, six patients developed solid tumors with a mean time of occurrence of 87 months (3–156) (4 pulmonary cancers, 1 breast cancer and 1 cervical carcinoma). Twelve patients (8 %) developed hematological malignancies. Six cases of MGUS (IgM) (4 %) and NHL (4 %) occurred with a mean time of 75 months (0–264) and 58 months (0–120), respectively. Four patients developed a diffuse large B-cell lymphoma and 2 patients a splenic marginal zone lymphoma (Table 2 for details). Among these 18 cases, 17 patients were chronic smokers. These data strongly lead us to consider PPBL as a premalignant state requiring a long-term follow-up.

**Table 1:** Characteristics and follow-up of 150 patients with PPBL

Age (years), Mean (min–max)	40 (18.9–66.2)		
Sex (M/F)	26 (17 %)/124 (83 %)		
Tobacco consumption	130/145 (90 %)		
Clinical presentation			
Lymph node(s)	10/108 (9 %)		
Splenomegaly	19/106 (18 %)		
Hepatomegaly	2/108 (2 %)		
Hemogram, Mean (min–max)			
White blood cells (10^9^/L)	12.8 (7–44.8)		
Hemoglobin (g/dL)	13.8 (10.1–16.9)		
Platelets (10^9^/L)	228 (83–380)		
Lymphocytosis (10^9^/L)	6.5 (2.2–41)		
Binucleated Lymphocytes (% of lymphocytes)	3.9 (1–40)		
IgM (g/L), Mean (min–max)	7.8 (2.17–20)		
HLA DR7 positive	40/52 (77 %)		
Multiparameter Flow Cytometry—Mean (min–max)			
CD19 (%)	50.4 (7–83)		
Cytogenetics		Diagnosis	Follow-up
+i(3)(q10) positive by karyotype		50/140 (36 %)	20/32 (63 %)
+i(3)(q10) positive by FISH		80/128 (63 %)	24/26 (92 %)
PCC positive		35/140 (25 %)	8/32 (25 %)
Chromosomal instability		76/140 (54 %)	31/32 (97 %)
Subsequent Malignancies	18/150 (12 %)		
MGUS	6/150 (4 %)		
Non-Hodgkin’s Lymphomas	6/150 (4 %)		
Solid tumors	6/150 (4 %)		

Clinical and biological data were collected from 27 centers. Median follow-up was 60 months (1–402) with unreached median overall survival

*MGUS* monoclonal gammopathy of undetermined significance

**Table 2:** Eighteen subsequent malignancies occurred in PPBL patients

Patients	Delay between PPBL and subsequent malignancy’s diagnosis	Type of malignancy	Follow-up
UPN36	38 months	DLBCL	56 months
UPN47	20 months	SMZL	+65 months
UPN57	92 months	DLBCL	99 months
UPN63	Diagnosis of PPBL and lymphoma was concomitant	DLBCL	+13 months
UPN71	77 months	SMZL	+86 months
UPN83	120 months	DLBCL	+131 months
UPN1	264 months	MGUS	+348 months
UPN10	144 months	MGUS	+148 months
UPN157	44 months	MGUS	+47 months
UPN118	Diagnosis of PPBL and MGUS was concomitant	MGUS	+36 months
UPN163	Diagnosis of PPBL and MGUS was concomitant	MGUS	+57 months
UPN105	Diagnosis of PPBL and MGUS was concomitant	MGUS	+42 months
UPN5	96 months	Mammary carcinoma	+272 months
UPN6	3 months	Pulmonary carcinoma	3 months
UPN70	22 months	Pulmonary carcinoma	+22 months
UPN86	132 months	Pulmonary carcinoma	+146 months
UPN160	114 months	Pulmonary carcinoma	112 months
UPN67	156 months	Cervical carcinoma	+181 months

Six patients developed solid tumors (4 pulmonary cancers, 1 breast cancer and 1 cervical carcinoma) and 6 patients hematological malignancies (diffuse large B-cell lymphoma (DLBCL) in 4 cases, splenic marginal zone lymphoma (SMZL) in 2 cases) and 6 patients monoclonal gammopathies of undetermined significance (MGUS) (IgM)

At diagnosis, CCA and FISH were performed in 140 and 128 patients, respectively. During the follow-up, CCA was performed in 32 patients (21 %). CCA and FISH detected no cytogenetic abnormality in 52/140 patients (37 %). Recurrent supernumerary isochromosome +i(3)(q10) was identified in 82/140 patients (59 %). PCC, arising from asynchronous mitotic activity in multinucleated cells, was observed concomitantly with +i(3)(q10) in 30/140 patients (21 %). By CCA, trisomy 8 and del(6q) were also detected either as recurrent abnormalities (2/140 and 5/140, respectively) or as non-recurrent abnormalities (9/140 and 4/140, respectively). Chromosomal instability was observed in 76/140 patients (54 %) and persisted in 31/32 patients (97 %) during follow-up.

**Fig. 1:**
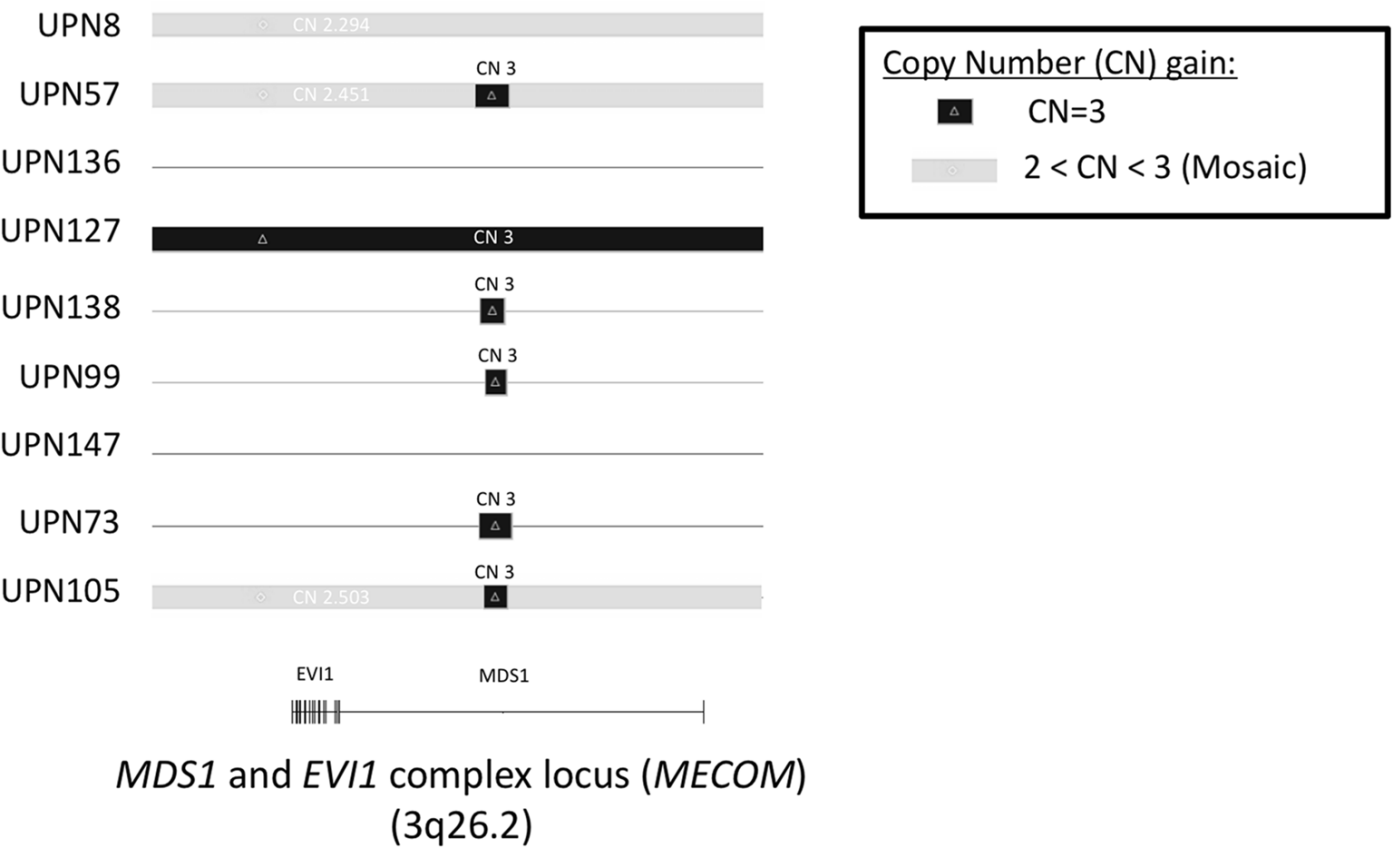
Schematic representation of region 3q26.2 corresponding to *MECOM* gene in CD19^+^ B-cells of 9 patients. We observed a common minimal amplified region of 28 kilobases (85 copy number markers) in 7 patients. This amplification is observed in all the CD19^+^ B-cells in 6 patients (copy number at 3, CN 3) and in a part of CD19^+^ B-cells in 1 patient (copy number between 2 and 3 revealing a mosaicism phenomenon)

To determine whether 3q could be implicated in PPBL pathogenesis, SNP arrays were performed in 10 patients (3 males, 7 females) with +i(3)(q10) in 9/10 patients (Table 3 for details). Written informed consents were obtained from the patients. The comparative analysis of sorted CD19^+^ and CD19^−^ cells revealed that CNAs were observed predominantly in CD19^+^ B-cells on 3q (Table 4) with mosaicism phenomenon in 3 patients. Genetic instability was observed in all cases and predominantly in CD19^+^ B-cells. We observed 143 recurrent CNAs with 129 gains (90 %) on 3q of B-cells (Table 5). Interestingly, we identified with a high frequency (7/9 patients) partial or complete amplification of one particular genomic region located in 3q26.2. The size of this common minimal amplified region was 28 kilobases (85 copy number markers) located in coding region of *MECOM* gene (Fig. 1). This amplification was not detected in two patients (UPN147 and UPN136). In one of them (UPN147), no +i(3)(q10) was detected by CCA and/or FISH. Unfortunately, due to the mosaicism phenomenon, with less than 20 % of B-cells presenting +i(3)(q10), gain in *MECOM* gene has not been confirmed yet by other molecular studies, such as quantitative PCR.

**Table 3:** Characteristics of the 10 patients analyzed by SNP arrays (UPN: Unique Patient Number)

Patient	Karyotype	PCC (%)	FISH +i(3)(q10) (%)
UPN8^b^	46–47,XX, +i(3)(q10) [3] /46,XX,del(2)(q22), −17, +mar [1] /45, X, −X [1] /46,XX [40]	Absent	Present (6 %)
UPN57^a^	47,XY, +i(3)(q10) [5] /48,XY, +i(3)(q10), +12 [01]/46,XY,t(14;18)(q32;q22)[01]/47,XY,t(11;14)(q13;q32), +mar [01]/46,XY,add(3)(p26) [1] /47,XY, +22[01]/49,XY, +i(3)(q10), +8, +mar[01]/46,XY [39]	Absent	Present (7 %)
UPN136^a^	46,XX [48]/PCC [2]	Present (4 %)	Present (4 %)
UPN71^c^	47,XX, +X,del(6)(q15q26)[01]/46,XX,del(6)(q15q26),der(6)t(6;6)(q21;q23)[08]/46,XX,del(1)(q12),der(14)t(1;14)(p22;q32)[02]/46,XX[09]	Absent	Present (2 %)
UPN127^b^	47,XY, +i(3)(q10) [3] /46,X,der(Y)t(Y;?)(q12;?) [3] /46,XY [12]	Absent	Present (12 %)
UPN138^a^	46,XX,del(6)(q21q24) [6] /46,XX,der(8)t(3;8)(q11;q11),der(17)t(17;?)(p11;?) [2] /46,XX,del(17)(p11) [2] /46,XX,t(1;6)(q24;q21) [1] /46,XX,der(14)t(14;?)(p25;?) [1] /46,XX,dup(3)(p13p26) [1] /46,XX,der(4)t(4;?)(p16;?) [1] /46,XX [26]	Absent	Present (11 %)
UPN99^a^	47,XX, +18 [2] /47,XX, +3 [1] /46,XX [37]/PCC [1]	Present (2 %)	Present (4 %)
UPN147^a^	46,XX [50]	Absent	Absent
UPN73^a^	46,XX [50]	Absent	Present (1.4 %)
UPN105^a^	46,XY [46]/46,XY [cp 4]	Absent	Present (3 %)

Depending on the quality of extracted DNA, we performed DNA arrays on ^a^both CD19^+^ and CD19^−^ cells in 7 patients, ^b^CD19^+^ cells in 2 patients and ^c^CD19^−^ cells in 1 patient. CCA and/or FISH detected +i(3)(q10) in 9/10 patients

*PCC* premature chromosome condensation

**Table 4:** Repartition of CNAs observed in CD19^−^ and CD19^+^ cells. CD19^+^ cells presented twice as many CNAs as CD19^−^ [83 CNAs (12–218) versus 42 (3–184)]

CD19−	CNAs—Total (gains/losses)								
Chromosome	UPN73	UPN71	UPN57	UPN136	UPN138	UPN99	UPN147	UPN105	Mean
1	2 (2/0)	0 (0/0)	4 (1/3)	1 (0/1)	3 (3/0)	1 (0/1)	0 (0/0)	17 (0/17)	3.5 (0.7/2.8)
2	3 (1/2)	1 (1/0)	0 (0/0)	2 (0/2)	2 (2/0)	0 (0/0)	0 (0/0)	24 (0/24)	4.0 (0.5/3.5)
3	2 (1/1)	0 (0/0)	3 (3/0)	0 (0/0)	0 (0/0)	0 (0/0)	0 (0/0)	8 (1/7)	1.6 (0.6/1.0)
3p	0 (0/0)	0 (0/0)	0 (0/0)	0 (0/0)	0 (0/0)	0 (0/0)	0 (0/0)	0 (0/0)	0.0 (0.0/0.0)
*3q*	*2 (1/1)*	*0 (0/0)*	*3 (3/0)*	*0 (0/0)*	*0 (0/0)*	*0 (0/0)*	*0 (0/0)*	*8 (1/7)*	*1.6 (0.6/1.0)*
4	0 (0/0)	0 (0/0)	0 (0/0)	2 (1/1)	0 (0/0)	0 (0/0)	1 (1/0)	30 (1/29)	4.1 (0.4/3.7)
5	0 (0/0)	1 (0/1)	1 (0/1)	0 (0/0)	1 (1/0)	0 (0/0)	0 (0/0)	15 (0/15)	2.3 (0.1/2.2)
6	0 (0/0)	0 (0/0)	0 (0/0)	0 (0/0)	1 (1/0)	0 (0/0)	0 (0/0)	11 (0/11)	1.5 (0.1/1.4)
7	0 (0/0)	0 (0/0)	0 (0/0)	0 (0/0)	1 (1/0)	0 (0/0)	1 (1/0)	12 (0/12)	1.8 (0.3/1.5)
8	1 (0/1)	0 (0/0)	0 (0/0)	1 (1/0)	3 (3/0)	0 (0/0)	0 (0/0)	6 (0/6)	1.4 (0.5/0.9)
9	0 (0/0)	4 (0/4)	3 (0/3)	0 (0/0)	4 (2/2)	1 (1/0)	0 (0/0)	7 (0/7)	2.4 (0.4/2.0)
10	1 (1/0)	1 (0/1)	1 (0/1)	0 (0/0)	1 (1/0)	0 (0/0)	1 (1/0)	7 (0/7)	1.5 (0.4/1.1)
11	0 (0/0)	2 (1/1)	0 (0/0)	0 (0/0)	2 (2/0)	0 (0/0)	1 (0/1)	10 (0/10)	1.9 (0.4/1.5)
12	2 (2/0)	0 (0/0)	1 (0/1)	0 (0/0)	1 (1/0)	0 (0/0)	0 (0/0)	8 (1/7)	1.5 (0.5/1.0)
13	0 (0/0)	0 (0/0)	2 (0/2)	0 (0/0)	1 (0/1)	0 (0/0)	0 (0/0)	8 (1/7)	1.4 (0.1/1.3)
14	0 (0/0)	0 (0/0)	0 (0/0)	0 (0/0)	0 (0/0)	0 (0/0)	0 (0/0)	6 (1/5)	0.8 (0.1/0.7)
15	2 (2/0)	1 (1/0)	1 (0/1)	2 (0/2)	2 (1/1)	0 (0/0)	1 (1/0)	4 (1/3)	1.6 (0.9/0.7)
16	1 (1/0)	0 (0/0)	0 (0/0)	1 (1/0)	3 (1/2)	0 (0/0)	0 (0/0)	1 (0/1)	0.8 (0.4/0.4)
17	0 (0/0)	0 (0/0)	1 (0/1)	0 (0/0)	1 (1/0)	0 (0/0)	0 (0/0)	4 (2/2)	0.8 (0.4/0.4)
18	0 (0/0)	0 (0/0)	0 (0/0)	0 (0/0)	1 (1/0)	0 (0/0)	0 (0/0)	1 (0/1)	0.2 (0.2/0.1)
19	0 (0/0)	1 (0/1)	0 (0/0)	0 (0/0)	0 (0/0)	1 (0/1)	1 (0/1)	1 (0/1)	0.5 (0.0/0.5)
20	1 (1/0)	0 (0/0)	0 (0/0)	0 (0/0)	1 (1/0)	0 (0/0)	0 (0/0)	0 (0/0)	0.3 (0.3/0.0)
21	1 (0/1)	1 (0/1)	1 (0/1)	0 (0/0)	0 (0/0)	0 (0/0)	0 (0/0)	1 (0/1)	0.5 (0.0/0.5)
22	0 (0/0)	0 (0/0)	0 (0/0)	0 (0/0)	0 (0/0)	0 (0/0)	1 (1/0)	1 (1/0)	0.3 (0.3/0.0)
X	1 (1/0)	7 (6/1)	13 (13/0)	1 (0/1)	25 (24/1)	0 (0/0)	2 (1/1)	1 (0/1)	6.2 (5.6/0.6)
Y	1 (1/0)	1 (1/0)	2 (0/2)	0 (0/0)	2 (2/0)	0 (0/0)	0 (0/0)	1 (0/1)	0.9 (0.5/0.4)
Total	18 (13/5)	20 (10/10)	33 (18/15)	10 (3/7)	55 (48/7)	3 (1/2)	9 (6/3)	184 (9/175)	41.5 (13.5/28)

CD19+	CNAs—total (gains/losses)									
Chromosome	UPN73	UPN8	UPN57	UPN136	UPN127	UPN138	UPN99	UPN147	UPN105	Mean
1	20 (19/1)	0 (0/0)	0 (0/0)	4 (0/4)	0 (0/0)	0 (0/0)	5 (4/1)	8 (1/7)	0 (0/0)	5.0 (3.6/1.4)
2	22 (22/0)	2 (1/1)	0 (0/0)	2 (0/2)	0 (0/0)	1 (0/1)	3 (3/0)	12 (1/11)	0 (0/0)	5.7 (3.9/1.8)
3	20 (19/1)	4 (4/0)	26 (25/1)	0 (0/0)	46 (46/0)	1 (1/0)	2 (2/0)	5 (0/5)	113 (113/0)	25.0 (24.2/0.8)
3p	11 (11/0)	0 (0/0)	1 (0/1)	0 (0/0)	0 (0/0)	0 (0/0)	0 (0/0)	0 (0/0)	0 (0/0)	1.8 (1.7/0.1)
*3q*	*9 (8/1)*	*4 (4/0)*	*25 (25/0)*	*0 (0/0)*	*46 (46/0)*	*1 (1/0)*	*2 (2/0)*	*5 (0/5)*	*113 (113/0)*	*23.2 (22.6/0.7)*
4	9 (9/0)	0 (0/0)	2 (0/2)	5 (1/4)	0 (0/0)	1 (1/0)	0 (0/0)	10 (1/9)	0 (0/0)	4.0 (2.3/1.7)
5	14 (13/1)	0 (0/0)	0 (0/0)	2 (0/2)	0 (0/0)	0 (0/0)	0 (0/0)	5 (0/5)	1 (0/1)	3.4 (2.4/1.0)
6	10 (10/0)	1 (1/0)	0 (0/0)	1 (0/1)	0 (0/0)	1 (1/0)	1 (1/0)	5 (1/4)	0 (0/0)	2.9 (2.3/0.6)
7	12 (12/0)	1 (0/1)	0 (0/0)	1 (0/1)	1 (0/1)	0 (0/0)	1 (1/0)	6 (1/5)	0 (0/0)	2.4 (1.6/0.8)
8	13 (11/2)	0 (0/0)	0 (0/0)	0 (0/0)	0 (0/0)	1 (0/1)	1 (1/0)	2 (0/2)	0 (0/0)	2.3 (1.8/0.5)
9	14 (14/0)	0 (0/0)	0 (0/0)	4 (0/4)	0 (0/0)	5 (0/5)	1 (1/0)	4 (1/3)	0 (0/0)	3.8 (2.4/1.4)
10	8 (8/0)	0 (0/0)	0 (0/0)	3 (0/3)	0 (0/0)	0 (0/0)	0 (0/0)	0 (0/0)	0 (0/0)	1.6 (1.2/0.4)
11	9 (9/0)	0 (0/0)	0 (0/0)	2 (0/2)	0 (0/0)	0 (0/0)	1 (1/0)	7 (0/7)	0 (0/0)	2.8 (1.8/1.0)
12	5 (5/0)	1 (0/1)	2 (0/2)	0 (0/0)	1 (1/0)	0 (0/0)	1 (1/0)	8 (1/7)	0 (0/0)	2.7 (1.6/1.1)
13	4 (4/0)	0 (0/0)	1 (0/1)	2 (0/2)	0 (0/0)	1 (0/1)	0 (0/0)	2 (0/2)	1 (1/0)	1.3 (0.7/0.6)
14	3 (3/0)	0 (0/0)	1 (0/1)	0 (0/0)	0 (0/0)	0 (0/0)	2 (2/0)	0 (0/0)	0 (0/0)	1.1 (1.0/0.1)
15	5 (5/0)	0 (0/0)	1 (1/0)	1 (0/1)	2 (0/2)	1 (0/1)	0 (0/0)	2 (1/1)	1 (1/0)	1.8 (1.2/0.6)
16	2 (2/0)	0 (0/0)	0 (0/0)	2 (0/2)	1 (1/0)	2 (0/2)	0 (0/0)	1 (1/0)	1 (0/1)	1.6 (1.0/0.6)
17	4 (4/0)	0 (0/0)	0 (0/0)	1 (0/1)	1 (1/0)	0 (0/0)	0 (0/0)	1 (1/0)	0 (0/0)	0.9 (0.8/0.1)
18	5 (5/0)	1 (1/0)	0 (0/0)	0 (0/0)	0 (0/0)	3 (3/0)	1 (1/0)	1 (0/1)	0 (0/0)	1.9 (1.8/0.1)
19	1 (1/0)	0 (0/0)	0 (0/0)	0 (0/0)	1 (0/1)	0 (0/0)	0 (0/0)	1 (0/1)	0 (0/0)	0.4 (0.2/0.2)
20	5 (5/0)	0 (0/0)	0 (0/0)	0 (0/0)	0 (0/0)	0 (0/0)	2 (2/0)	1 (1/0)	0 (0/0)	1.2 (1.2/0.0)
21	1 (0/1)	0 (0/0)	0 (0/0)	1 (0/1)	0 (0/0)	1 (0/1)	0 (0/0)	2 (0/2)	0 (0/0)	0.6 (0.0/0.6)
22	0 (0/0)	0 (0/0)	0 (0/0)	0 (0/0)	0 (0/0)	0 (0/0)	0 (0/0)	1 (1/0)	0 (0/0)	0.3 (0.3/0.0)
X	28 (27/1)	1 (0/1)	1 (1/0)	7 (5/2)	2 (2/0)	5 (4/1)	1 (1/0)	5 (3/2)	0 (0/0)	7.0 (6.2/0.2)
Y	4 (4/0)	1 (1/0)	0 (0/0)	6 (6/0)	0 (0/0)	14 (14/0)	2 (2/0)	0 (0/0)	0 (0/0)	3.0 (3.0/0.0)
Total	218 (211/7)	12 (8/4)	34 (27/7)	44 (12/32)	55 (51/4)	37 (24/13)	24 (23/1)	89 (15/74)	117 (115/2)	82.7 (66.6/16.1)

28 % of CNAs (0–97 %) were located on 3q in CD19^+^ cells compared to 5 % (0–11 %) in CD19^−^ cells (data not shown)

**Table 5:** Recurrent Copy Number Aberrations (CNA) in CD19^+^ B-cells. 143 CNA had been observed

Chr	Cytoregion	Recurrence	Recurrence including mosaicism	CN state	Gene	Minimal common size (kbp)	Genic region: total (T) Exonic (E) Intronic (I)	CNA reported in DGV
1	p33	2	2	Loss	FAF1	31	I	No
1	p32.2	2	2	Gain	C1orf168	50.2	E/I	No
2	p23.2	2	2	Gain	ALK	62	E/I	Yes
2	q21.2–q21.3	2	2	Gain	MGAT5	72	E/I	No
3	p24.2	2	2	Gain	THRB	55.7	I	No
3	q11.2	2	3	Gain	LOC255025	50	E/I	No
3	q12.2	2	3	Gain	ABI3BP	143	E/I	Yes
3	q13.13	2	3	Gain	DZIP3	10.6	I	No
3	q13.31	2	3	Gain	ZBTB20	39	I	No
3	q13.31	2	3	Gain	GAP43	399	T	No
3	q13.31	2	3	Gain	LSAMP	53	I	No
3	q13.33	2	3	Gain	TMEM39A	176	T	No
3	q13.33	2	3	Gain	KTELC1	176	T	No
3	q13.33	2	3	Gain	C3orf1	176	T	No
3	q13.33	2	3	Gain	CD80	176	T	No
3	q13.33	2	3	Gain	ADPRH	176	T	No
3	q21.1	2	3	Gain	HSPBAP1	101	E/I	No
3	q21.1	2	4	Gain	DIRC2	101	T	No
3	q21.1	2	4	Gain	LOC100129550	101	T	Yes
3	q21.1	2	4	Gain	SEC22A	114	T	No
3	q21.1	2	4	Gain	PTPLB	125	T	No
3	q21.1	3	4	Gain	MYLK	70	E/I	No
3	q21.1	2	4	Gain	CCDC14	121	E/I	Yes
3	q21.2	2	4	Gain	KALRN	169	T	No
3	q21.2	2	4	Gain	UMPS	169	T	No
3	q21.2	2	4	Gain	ZNF148	164	E/I	Yes
3	q21.2	2	4	Gain	ALDH1L1	171	E/I	No
3	q21.3	3	4	Gain	TXNRD3IT1	299	E/I	No
3	q21.3	3	4	Gain	CHCHD6	299	E/I	No
3	q21.3	2	4	Gain	KLHDC6	95	T	No
3	q21.3	2	4	Gain	RUVBL1	211	E/I	Yes
3	q21.3	2	4	Gain	EEFSEC	211	E/I	Yes
3	q21.3	2	4	Gain	GATA2	76	E/I	Yes
3	q21.3	3	4	Gain	LOC90246	76	T	Yes
3	q21.3	2	4	Gain	C3orf27	120.7	T	Yes
3	q21.3	2	4	Gain	TMCC1	268	T	No
3	q21.3	2	4	Gain	COL6A4P2	131	T	Yes
3	q22.1	2	4	Gain	MRPL3	62	E/I	Yes
3	q22.1	2	4	Gain	SNORA58	62	T	Yes
3	q22.1	3	5	Gain	CPNE4	46	I	Yes
3	q22.1	2	4	Gain	CPNE4	155	E/I	Yes
3	q22.1	2	4	Gain	TMEM108	120	I	Yes
3	q22.1	2	4	Gain	TOPBP1	69	E/I	No
3	q22.1	2	4	Gain	RYK	225	T	No
3	q22.1	2	4	Gain	ANAPC13	197	T	Yes
3	q22.1	2	4	Gain	CEP63	197	T	Yes
3	q22.2	2	4	Gain	EPHB1	144	E/I	No
3	q22.2	2	4	Gain	PPP2R3A	85	E/I	No
3	q22.3	2	4	Gain	SOX14	925	T	No
3	q22.3	3	4	Gain	CLDN18	121	T	Yes
3	q22.3	2	4	Gain	ARMC8	77	E/I	Yes
3	q22.3	2	4	Gain	TXNDC6	77	E/I	Yes
3	q22.3	2	4	Gain	ESYT3	202.7	E/I	No
3	q22.3	2	4	Gain	CEP70	202.7	T	No
3	q22.3	2	4	Gain	FAIM	202.7	T	No
3	q22.3	2	4	Gain	PIK3CB	202.7	E/I	No
3	q22.3	3	4	Gain	LOC729627	193	T	No
3	q22.3	3	4	Gain	LOC389151	193	T	No
3	q22.3	3	4	Gain	FLJ46210	193	T	No
3	q22.3	3	4	Gain	BPESC1	193	T	No
3	q22.3	2	4	Gain	PISRT1	319	T	No
3	q23	2	4	Gain	MRPS22	89	E/I	No
3	q23	2	4	Gain	COPB2	89	T	No
3	q23	3	4	Gain	NMNAT3	277.8	E/I	No
3	q23	4	5	Gain	CLSTN2	46	I	Yes
3	q23	2	4	Gain	TRIM42	443	T	Yes
3	q23	2	4	Gain	SLC25A36	443	T	Yes
3	q24	2	4	Gain	SLC9A9	138	E/I	Yes
3	q24	2	4	Gain	PLSCR4	47	E/I	No
3	q24	2	4	Gain	PLSCR5	69	T	No
3	q24	2	4	Gain	AGTR1	194	E/I	No
3	q25.1	2	4	Gain	P2RY13	74	E/I	No
3	q25.1	2	4	Gain	MED12L	74	E/I	No
3	q25.1	2	4	Gain	P2RY13	74	T	No
3	q25.2	2	4	Gain	SGEF	364	E/I	Yes
3	q25.2–q25.31	3	4	Gain	MME	87.4	E/I	No
3	q25.32	2	4	Gain	VEPH1	78	E/I	Yes
3	q25.32	2	4	Gain	C3orf55	78	E/I	No
3	q25.32	3	4	Gain	MLF1	50	E/I	No
3	q26.1	2	4	Gain	C3orf57	120.6	E/I	No
3	q26.1	2	4	Gain	OTOL1	120.6	T	No
3	q26.1	3	4	Gain	SI	747	T	No
3	q26.1	3	4	Gain	BCHE	329	E/I	No
3	q26.1	2	4	Gain	ZBBX	307	T	No
*3*	*q26.2*	*6*	*7*	*Gain*	*MDS1*	*28*	*E/I*	*No*
3	q26.2	2	4	Gain	TERC	59	T	Yes
3	q26.2	2	4	Gain	ARPM1	59	T	Yes
3	q26.2	2	4	Gain	MYNN	59	T	Yes
3	q26.2	2	4	Gain	LRRC34	59	E/I	Yes
3	q26.2	3	5	Gain	TNIK	31	E/I	No
3	q26.31	2	4	Gain	NLGN1	125	I	Yes
3	q26.31	2	4	Gain	NLGN1	64	E/I	No
3	q26.31	2	4	Gain	NAALADL2	113	E/I	Yes
3	q26.32	2	4	Gain	TBL1XR1	60	E/I	No
3	q26.32	2	4	Gain	KCNMB2	121	E/I	No
3	q26.33	2	4	Gain	USP13	59	E/I	No
3	q26.33	2	4	Gain	PEX5L	81	E/I	No
3	q26.33	2	4	Gain	CCDC39	118	E/I	Yes
3	q27.1	2	4	Gain	YEATS2	112	E/I	No
3	q27.1	2	4	Gain	MAP6D1	112	T	No
3	q27.1	2	4	Gain	PARL	112	E/I	No
3	q27.2	2	4	Gain	VPS8	218	E/I	No
3	q27.2	2	4	Gain	ETV5	157	T	No
3	q27.2	2	4	Gain	DGKG	157	E/I	No
3	q27.3	2	4	Gain	CRYGS	110	E/I	No
3	q27.3	2	4	Gain	TBCCD1	110	T	No
3	q27.3	2	4	Gain	DNAJB11	110	T	No
3	q27.3	2	4	Gain	AHSG	110	T	Yes
3	q27.3	2	4	Gain	FETUB	110	E/I	Yes
3	q27.3	2	4	Gain	ST6GAL1	46	E/I	Yes
3	q27.3	2	4	Gain	MASP1	428	E/I	No
3	q27.3	3	4	Gain	RTP4	148	T	No
3	q27.3	2	4	Gain	SST	428	T	No
3	q27.3	2	4	Gain	FLJ42393	191	T	Yes
3	q28	3	4	Gain	LPP	191	E/I	Yes
3	q28	2	4	Gain	TP63	142	E/I	No
3	q28	2	4	Gain	CLDN1	203	T	No
3	q28	2	4	Gain	CLDN16	203	T	No
3	q28	2	4	Gain	TMEM207	203	T	No
3	q29	2	4	Gain	C3orf59	396	E/I	No
3	q29	2	4	Gain	MGC2889	396	T	Yes
3	q29	2	4	Gain	HRASLS	396	T	Yes
3	q29	2	4	Gain	ATP13A5	396	E/I	No
3	q29	2	4	Gain	ATP13A4	158	E/I	Yes
3	q29	2	4	Gain	OPA1	158	T	Yes
3	q29	2	4	Gain	GP5	97	E/I	No
3	q29	2	4	Gain	ATP13A3	97	T	Yes
3	q29	2	4	Gain	TM4SF19	87	E/I	Yes
3	q29	2	4	Gain	UBXN7	87	E/I	Yes
3	q29	2	4	Gain	DLG1	240	E/I	Yes
3	q29	2	4	Gain	FYTTD1	50	T	Yes
3	q29	2	4	Gain	LRCH3	50	E/I	Yes
3	q29	2	4	Gain	RPL35A	91	E/I	Yes
3	q29	2	4	Gain	IQCG	91	E/I	Yes
3	q29	2	4	Gain	LMLN	91	T	Yes
4	q13.3	2	2	Gain	SLC4A4	46	E/I	No
11	p15.1	2	2	Gain	NELL1	43	I	No
14	q13.1	2	2	Gain	NPAS3	43	I	Yes
16	p11.1	2	2	Gain	LOC283914	277	T	Yes
21	p11.2–p11.1	2	3	Loss	TPTE	107	T	Yes
X	p22.33	3	3	Gain	DHRSX	31	E/I	Yes
X	q12	2	2	Gain	EDA2R	91	E/I	Yes
Y	q11.21	2	2	Gain	USP9Y	60	E/I	No

129 gains concerned the long arm of chromosome 3 (3q). 123 gains concerned gene coding regions. 75 CNA did not include previously reported polymorphism (Database of Genomic Variants, DGV). Gain of one exon of *MDS1* (part of *MECOM* gene) was recurrently observed in 7 patients (including mosaicism phenomenon)

*Chr* chromosome, *Recurrence* number of patients with the same CNA, *CN state* Copy Number state, gain or loss

## Discussion

Similar to the described link between aneuploidy, genetic instability and the development of human cancers [11, 12], supernumerary isochromosome 3q could be the cause of chromosomal instability observed in PPBL. Transfer of isochromosome 3q into myoblast cell line caused abnormal cytokinesis, centrosome amplification, aneuploidy and abolished G1 arrest following DNA damage. These observations might be related to an increasing expression of *ATR* gene located on 3q [13]. Moreover, isochromosome 3q has been implicated in the progression of cervical carcinomas, where cells exhibiting either tetrasomy or aneusomy for chromosomes 3 and 17 increased significantly with disease progression [13–16]. Supernumerary isochromosome 3q could explain binucleated lymphocytes and chromosomal instability observed in PPBL. *MECOM* abnormalities, particularly the overexpression of *EVI1*, have been described in the pathogenesis of myeloid neoplasm such as acute myeloid leukemia and myelodysplastic syndrome, especially concerning cell-cycle disorders [17–21]. Furthermore, as observed by Stein et al., *EVI1* activation could lead to genetic instability [22]. Even if it has never been observed in lymphoid neoplasm, the potential implication of *MECOM* in PPBL has to be elucidated.

The link between PPBL and subsequent malignancies remains unclear and the role of tobacco is probably dominant. Majority of our patients (17/18) with subsequent malignancies were chronic smokers. We reported recently a detailed description of 2 heavy smokers patients with subsequent malignancies, UPN57 and UPN71 [23]. Tobacco use is a recognized risk factor in the development of solid tumor, such as pulmonary cancer, and also lymphoma [24]. Therefore, in PPBL, where tobacco consumption is frequent (90 % of our cohort of 150 patients), smoking could represent a confounding factor in interpreting the link between PPBL and subsequent malignancies.

Isochromosome 3q has been described in cell-cycle deregulation, chromosomal instability and progression of cervical cancers. Our cytogenetic and clinical observations lead us to hypothesize that isochromosome 3q in B-cells plays a key role in the physiopathology and evolution of PPBL. Although isochromosome 3q has not been yet identified in tumor cells of subsequent malignancies [23], it could be implicated in chromosomal and genomic instability. This genomic instability could be part of a multi-step process leading to the emergence of a malignant B lymphoproliferation. *MECOM* gene could be a good candidate to explain these observations and remains to be explored.

## Availability of data and materials

All raw data are available from the authors upon request.
